# Thermal and Kinetic Analysis of Benzimidazole Derivatives: Fenbendazole, Mebendazole, and Flubendazole

**DOI:** 10.3390/molecules31061005

**Published:** 2026-03-17

**Authors:** Adriana Ledeți, Ramona-Daniela Pârvănescu, Amalia Ridichie, Titus Vlase, Oana Suciu, Ovidiu Ghirlea, Marius Murariu, Carmen Tomoroga, Sebastian Simu, Ionuț Ledeți, Cristina Maria Trandafirescu

**Affiliations:** 1 Advanced Instrumental Screening Center, Faculty of Pharmacy, “Victor Babeș” University of Medicine and Pharmacy, Eftimie Murgu Square No. 2, 300041 Timisoara, Romania; afulias@umft.ro (A.L.); amalia.ridichie@umft.ro (A.R.); axente.carmen@umft.ro (C.T.); simu.sebastian@umft.ro (S.S.); 2 Research Center for Experimental Pharmacology and Drug Design, Faculty of Pharmacy, “Victor Babeș” University of Medicine and Pharmacy, Eftimie Murgu Square No. 2, 300041 Timisoara, Romania; ramona.parvanescu@umft.ro (R.-D.P.); trandafirescu.cristina@umft.ro (C.M.T.); 3 Research Centre for Thermal Analysis in Environmental Problems, West University of Timisoara, Pestalozzi Street 16, 300115 Timisoara, Romania; titus.vlase@e-uvt.ro; 4 Faculty of Medicine, “Victor Babeș” University of Medicine and Pharmacy, Eftimie Murgu Square 2, 300041 Timisoara, Romania; suciu.oana@umft.ro (O.S.); ovidiu.ghirlea@umft.ro (O.G.); murariu.marius@umft.ro (M.M.)

**Keywords:** benzimidazole derivatives, fenbendazole, mebendazole, flubendazole, thermal analysis, kinetic parameters

## Abstract

This study presents a comparative thermal and kinetic analysis of three benzimidazole derivatives used in the pharmaceutical field: fenbendazole, mebendazole, and flubendazole. The investigations were carried out using thermoanalytical methods, including thermogravimetric analysis (TGA) and differential scanning calorimetry (DSC), in order to evaluate thermal stability, decomposition stages, and to calculate kinetic parameters. The obtained data were processed using isoconversional methods (Ozawa–Flynn–Wall, and Friedman) and non-parametric method (NPK) to determine activation energies and degradation mechanisms. The results revealed significant differences among the three compounds regarding their thermal stability and decomposition behavior, influenced by molecular structure and aromatic substituents. Furthermore, the comparative analysis provides valuable insights for optimizing technological processes, assessing stability in pharmaceutical formulations, and expanding research on the therapeutic potential of these compounds, including in oncological studies. Overall, the study contributes to a deeper understanding of the relationship between chemical structure and thermal stability in benzimidazole derivatives.

## 1. Introduction

The three active pharmaceutical ingredients selected for thermoanalytical and kinetic investigation are fenbendazole, mebendazole, and flubendazole. These benzimidazole derivatives represent a class of compounds with potent antiparasitic activity, widely used in both veterinary and human medicine [1]. They share a common mechanism of action based on selective binding to β-tubulin in parasite cells, inhibiting microtubule polymerization. The disruption of microtubule formation impairs intracellular transport, glucose uptake, and energy metabolism, ultimately leading to glycogen depletion and parasite death through inhibition of ATP production [2,3]. These compounds are of significant pharmaceutical importance due to their broad-spectrum activity, favorable tolerability, versatility in medical applications, and relevance to public health. Moreover, recent studies suggest that fenbendazole, traditionally used as an anthelmintic agent, may exhibit potential anticancer properties by interfering with microtubule dynamics in malignant cells, thereby inhibiting mitosis, suppressing tumor proliferation, and promoting apoptosis [4,5,6].

**Figure 1:**
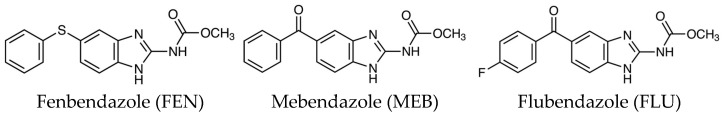
The chemical structures of the three benzimidazole structures.

Fenbendazole, mebendazole, and flubendazole share a benzimidazole core structure characterized by a fused benzene–imidazole ring system, but differ in their side-chain substitutions, which influence their pharmacological properties and pharmacological activity. The chemical structures for the three active substances are presented in Figure 1.

In the pharmaceutical field, thermal and kinetic analysis are two complementary and essential tools for the characterization of active substances and their pharmaceutical products [1,2,3]. Thermal analysis represents a set of techniques that investigate the physico-chemical changes of an API as a function of temperature, providing information about phase transitions, thermal stability, mass loss, or interactions with auxiliary substances used in the pharmaceutical industry. The data obtained from thermal analysis can be correlated with kinetic analysis, which examines the rate and mechanism of degradation or transformation processes [4], determining important quantitative parameters such as activation energy, pre-exponential factor, and reaction order [5].

The combination of these two types of analyses enables the prediction of substance behavior under storage conditions, optimization of technological processes, and predictability of shelf life [6], playing a crucial role in ensuring the quality, safety, and efficacy of pharmaceutical products.

In pharmaceutical research, thermal analysis [7] provides experimental data (e.g., melting temperatures, mass losses, phase transitions), while kinetic analysis [8] converts these data into quantitative parameters (reaction rates, activation energies, mechanisms). Together, these two methods enable a comprehensive evaluation of the stability and processability of active substances and their pharmaceutical formulations, thereby supporting the development of safe, effective, and high-quality pharmaceutical products [9].

## 2. Results and Discussion

### 2.1. Thermal Analysis

Thermal analysis was performed for the three active substances—fenbendazole, mebendazole, and flubendazole—in order to evaluate their thermal stability, decomposition stages, and degradation behavior as a function of molecular structure. The experiments were carried out using thermogravimetric analysis (TGA) and differential scanning calorimetry (DSC) at different heating rates (β = 7, 12, 15, and 20 °C min^−1^) in a synthetic air atmosphere, over a temperature range between 30 °C and 500 °C.

**Figure 2:**
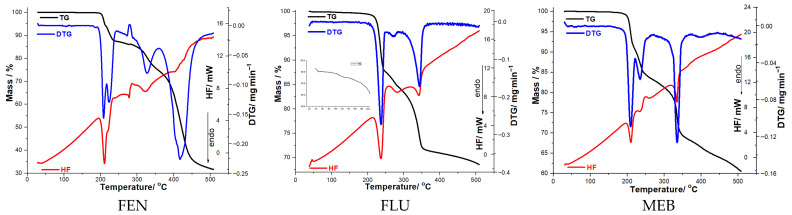
Thermoanalytical curves for the three active pharmaceutical ingredients.

The TGA curves revealed an initial mass loss stage associated with the removal of adsorbed water molecules (only in the case of FLU), followed by one or more main stages of thermal decomposition. For each compound, the onset and end temperatures of each mass-loss stage, as well as the corresponding mass change (Δm), were determined (see Table 1). The overlapping thermoanalytical curves for the three active substances are presented in Figure 2.

The results showed notable differences among the three benzimidazole derivatives. Flubendazole exhibited the highest thermal stability, followed by fenbendazole and mebendazole, a trend correlated with the presence and nature of aromatic substituents in their molecular structures. The HF curves confirmed these observations, displaying endothermic processes (melting) consistent with literature data [10,11,12] and exothermic events corresponding to decomposition.

The thermoanalytical profiles of fenbendazole, flubendazole, and mebendazole reveal multi-step thermal decomposition behavior, each characterized by distinct mass-loss events and corresponding DTG and HF signals. FEN exhibits three major degradation steps between ~197–500 °C, with significant mass losses of 13.58%, 12.20%, and 41.83%, accompanied by DTG peaks at 209–222 °C, 328 °C, and 404 °C, and multiple HF endo/exothermic transitions, indicating complex structural breakdown. FLU also shows a three-step degradation pattern, but with lower initial mass losses (11.64% and 3.56%) and DTG maxima occurring at slightly lower temperatures (238 °C, 272 °C, and 343 °C), suggesting improved thermal stability in the early stages compared to FEN. MEB displays a thermal behavior similar in pattern but overall, more gradual, with relatively moderate mass losses (8.41%, 8.63%, and 21.42%) and DTG peaks at 210 °C, 236 °C, and 335 °C; the HF profile shows several transitions associated with melting, recrystallization, and decomposition.

Overall, the data demonstrate that among the three benzimidazole derivatives, fenbendazole undergoes the most intense and highest-temperature degradation, while mebendazole exhibits the most moderate and progressive mass-loss profile.

**Figure 3:**
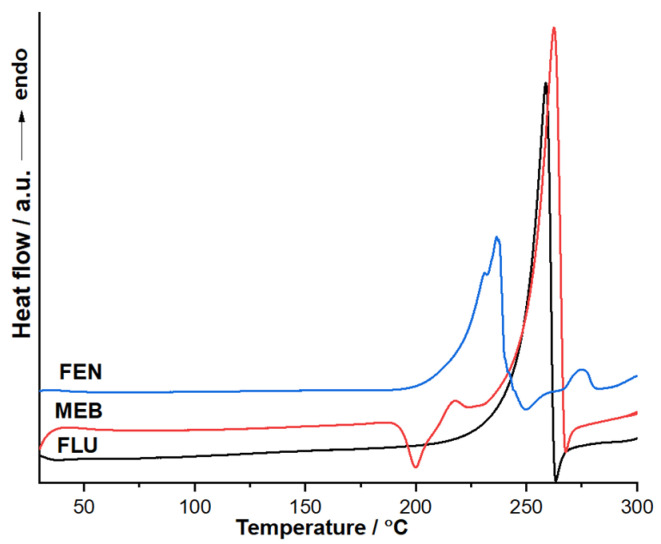
DSC curves for the three active pharmaceutical ingredients.

Differential scanning calorimetry (DSC) was used to analyze all three active substances (see Figure 3). Flubendazole exhibits a sharp and intense endothermic peak at approximately DSC_max_ = 258 °C, which can be attributed to its solid–liquid transition (Δ_fus_H = 158.5 J g^−1^). The narrow peak shape and high intensity suggest the fusion of a well-defined crystalline phase with good purity. No additional thermal events are observed prior to melting, indicating the absence of solid–solid polymorphic transitions within the investigated temperature range. Mebendazole shows a more complex thermal behavior. An exothermic event is observed around 195–205 °C (DSC_max_ = 200 °C), which may correspond to a solid-state transition or crystallization process. This is followed by a strong endothermic event (i.e., melting) at approximately DSC_max_ = 262.5 °C. The presence of the additional thermal event suggests possible polymorphism or structural rearrangement before melting.

Overall, both compounds display high melting temperatures, consistent with their rigid benzimidazole structures and strong intermolecular interactions. The sharper melting peak observed for flubendazole indicates a more homogeneous crystalline phase compared to mebendazole. The DSC curve corresponding to fenbendazole (FEN) reveals a distinct thermal profile compared to flubendazole (FLU) and mebendazole (MEB). In the temperature range of approximately 50–200 °C, the FEN curve remains relatively stable, showing no significant endothermic or exothermic events. This indicates the absence of dehydration processes, polymorphic transitions, or solid–solid phase transformations within this interval, suggesting good solid-state stability under moderate thermal conditions. A pronounced endothermic peak is observed at approximately DSC_max_ = 236 °C (Δ_fus_H = 99.05 J g^−1^), which can be attributed to the solid–liquid transition of FEN. The sharp and well-defined nature of this peak suggests a high degree of crystallinity and chemical purity. Immediately following the melting event, a rapid change in heat flow is detected, indicating the onset of thermal degradation.

Although a polymorphic transformation of mebendazole around ~200 °C has been reported in the literature [13], in our experiments the DSC exotherm observed at ~200–210 °C coincides with the onset of mass loss in TG. This indicates that, under the present conditions (10 °C min^−1^, dynamic atmosphere), the transformation in this temperature region is not purely a physical solid–solid transition but is overlapped by an incipient chemical process (early decomposition and/or release of volatile species). Therefore, while a polymorphic contribution cannot be excluded, the combined DSC–TG evidence supports assigning the event to a coupled transformation process.

### 2.2. Kinetic Analysis

**Figure 4:**
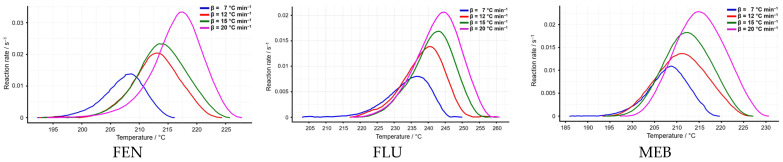
The reaction progress vs. temperature at selected heating rates for the three.

The temperature intervals selected for the kinetic analysis show a consistent shift toward higher values with increasing heating rates for all three benzimidazoles. Flubendazole exhibits the narrowest variation, with the process occurring between 203–261 °C across all heating rates, indicating relatively stable thermal behavior (see Figure 4).

Fenbendazole displays slightly lower onset temperatures (192–227 °C), while mebendazole shows the lowest initial temperatures (187–231 °C), suggesting that its degradation process begins earlier than in FLU and FEN. Overall, the systematic increase in temperature range with heating rate reflects the expected kinetic compensation effect, while the relative ordering of the onset temperatures (MEB < FEN < FLU) highlights subtle differences in thermal reactivity among the three compounds (see Table 2). In conclusion, the temperature intervals used for kinetic evaluation show a consistent shift to higher values with increasing heating rate, with mebendazole initiating degradation at the lowest temperatures, followed by fenbendazole and flubendazole, reflecting their relative thermal reactivity.

**Figure 5:**
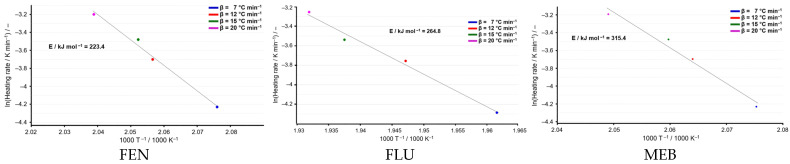
ASTM E698 kinetic plots for the studied samples.

The ASTM E698 method was applied for the first time to evaluate the kinetic behavior of the three active pharmaceutical substances—fenbendazole, flubendazole, and mebendazole—providing a reliable approach for determining their kinetic parameters based on non-isothermal thermal analysis. The ASTM E698 plots (Figure 5) show a good linear correlation between ln(β/T^2^) and 1000/T for all three active substances, indicating that the thermal decomposition processes follow Arrhenius-type behavior and that the ASTM E698 method is applicable in each case. From a comparative perspective, clear differences can be observed in the calculated activation energies. FEN exhibits the lowest activation energy (*E_a_* = 223.4 kJ·mol^−1^), suggesting a lower thermal stability and a higher susceptibility to thermal degradation. FLU shows an intermediate activation energy (*E_a_* = 264.8 kJ·mol^−1^), indicating improved thermal resistance compared to FEN. In contrast, MEB presents the highest activation energy (*E_a_* = 315.4 kJ·mol^−1^), reflecting the greatest thermal stability among the three compounds. The increasing activation energy from FEN to FLU and MEB suggests that structural differences between the molecules significantly influence their thermal behavior.

**Figure 6:**
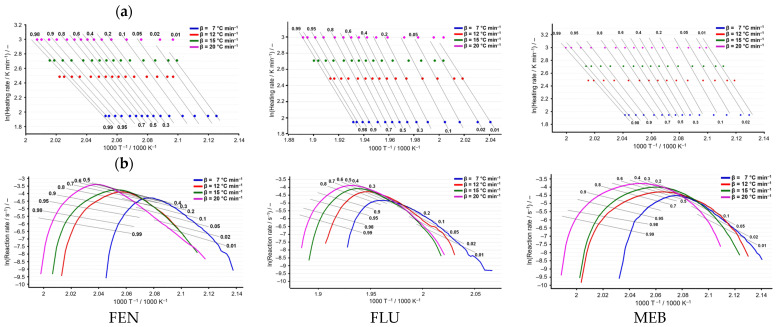
Isoconversional plots for the kinetic study of the decomposition of the three APIs: (**a**) the plots corresponding to Flynn–Wall–Ozawa method and (**b**) the Friedman plots.

The Flynn–Wall–Ozawa (see Figure 6a) and Friedman (see Figure 6b) isoconversional analyses revealed a good linearity of the corresponding plots for flubendazole, fenbendazole, and mebendazole at selected conversion degrees. The activation energy values, calculated from the slopes of the log β versus 1/T plots and ln(dα/dt) versus 1/T, showed comparable trends for both kinetic methods, highlighting differences in the thermal decomposition behavior of the three compounds.

**Figure 7:**
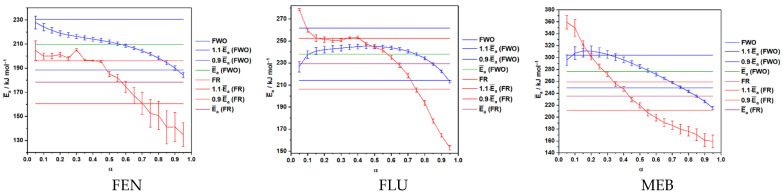
Variation of the activation energy with conversion degree (α), evaluated by two isoconversional methods, and the average *E_a_* values obtained for FEN, MEB, and FLU. The blue and red curves with symbols represent the *E_a_* values obtained by the FWO and FR methods, respectively. The horizontal blue and red lines correspond to ±10% variation limit of average *E_a_* (1.1·*E_a_* and 0.9·*E_a_*), respectively, while the solid horizontal green and purple lines indicate the average *E_a_* values for each method (green for FWO and purple for FR).

The activation energy profiles obtained using the FWO and FR isoconversional methods reveal distinct thermal decomposition behaviors for the three benzimidazole derivatives. For all compounds, *E_a_* shows moderate variation with the conversion degree (α), indicating that their degradation is a multi-step process rather than a single-stage reaction. MEB exhibits the highest activation energies across the entire α range, reflecting superior thermal stability, while FEN consistently displays the lowest values, confirming it as the least thermally resistant of the three. FLU presents intermediate *E_a_* values with relatively small fluctuations, suggesting a more uniform degradation pathway. Additionally, FR values are generally lower than the corresponding FWO values, which is consistent with the known tendency of Friedman’s differential method to provide slightly lower *E_a_* estimates due to its higher sensitivity to experimental noise. Overall, the trends observed across α reinforce that the decomposition mechanisms of these APIs evolve with conversion, highlighting the importance of applying model-free isoconversional approaches to accurately characterize their thermal behavior (see Figure 7).

In the case of FEN, the activation energy (*E_a_*) exhibited a gradual decrease with increasing conversion. The FWO method yielded a smooth decline from approximately 225 to 190 kJ mol^−1^, whereas the FR method determined values ranging from ~205 to ~140 kJ mol^−1^, accompanied by higher variability. Despite quantitative differences between the two approaches, both clearly show a monotonic reduction of *E_a_* across the conversion interval. For the FLU, *E_a_* displayed a non-monotonic trend. The FWO curves indicated a slight increase in *E_a_* at low α (0.05–0.3), followed by a steady decrease up to α = 1. A similar pattern was observed for the FR method, although with larger fluctuations and a sharper drop at high α values. The intermediate range (α ≈ 0.3–0.6) showed a relatively stable *E_a_* plateau for both methods. MEB showed the highest activation energies among the three active substances. Initial *E_a_* values exceeded 330 kJ mol^−1^ according to FWO, decreasing continuously to roughly 220 kJ mol^−1^ at α = 1. The FR method revealed an even steeper decline. Both approaches indicate substantial changes in the apparent activation energy throughout the decomposition process (see Table 3).

The reported uncertainties correspond to the standard deviation of the mean activation energy values calculated from the *E_a_* values obtained at different conversion degrees (α). Although the individual *E_a_* values exhibit relatively small point-to-point deviations, the variation of activation energy over the conversion range leads to a higher dispersion and consequently to a larger uncertainty associated with the calculated mean value.

**Figure 8:**
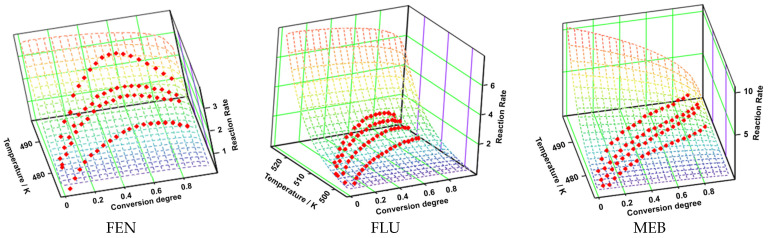
Three-dimensional surfaces describing the transformation rate for the three APIs, generated using the NPK method.

The non-parametric kinetic (NPK) method provides model-free activation energies by isolating the kinetic signal from the thermogravimetric data without assuming a reaction model. Figure 8 presents the three-dimensional transformation rate surfaces of FEN, FLU, and MEB as a function of temperature and conversion degree, generated using the NPK method. For all investigated APIs, the reaction rate exhibits a strong dependence on both temperature and conversion degree, indicating a complex kinetic behavior. The experimentally derived rate points are in good agreement with the NPK-reconstructed surfaces, confirming the reliability of the applied non-parametric approach.

An increase in temperature leads to a systematic increase in the transformation rate for all three compounds, while the rate does not vary monotonically with conversion degree. Although all three investigated active pharmaceutical ingredients (APIs) exhibit qualitatively similar trends in the evolution of the transformation rate, significant quantitative differences can be observed among them. MEB is characterized by the highest transformation rates as well as by the most pronounced sensitivity to temperature variations, indicating a strongly thermally activated process. In contrast, FLU displays lower transformation rates and a narrower rate distribution over the entire conversion degree range, suggesting a slower transformation that may be limited by structural or diffusional factors. FEN exhibits an intermediate behavior, being characterized by moderate transformation rates and a relatively uniform dependence of the transformation rate on the degree of conversion.

These differences can be attributed to intrinsic variations in the solid-state properties of the analyzed compounds, such as crystalline structure, thermal stability, or intermolecular interactions. At the same time, the obtained results highlight the usefulness and robustness of the non-parametric kinetic (NPK) method as an appropriate tool for the comparative analysis of the kinetic behavior of different active pharmaceutical ingredients.

The values in the table represent the average activation energies over the conversion range, along with the contributions of individual kinetic processes (λ), which reflect the weight of each step in the overall decomposition. The data obtained from the application of the NPK kinetic method are presented in the following table (see Table 4).

The non-parametric kinetic (NPK) analysis reveals that the thermal decomposition of fenbendazole (FEN), flubendazole (FLU), and mebendazole (MEB) proceeds through two distinct kinetic processes, each characterized by specific activation energies, reaction models, and contributions to the overall mass loss.

For FEN, the primary decomposition step (Process 1), accounting for λ = 82.8%, exhibits an activation energy of 212.4 ± 9.2 kJ·mol^−1^ and is best described by a reaction model of the form (1−x)^1/10^, suggesting a diffusion-controlled or decelerating reaction mechanism. The relatively high pre-exponential factor (A ≈ 10^23^ min^−1^) indicates a high frequency of successful molecular collisions once the activation barrier is surpassed. The secondary process (λ = 13.8%) shows a lower activation energy (179.8 ± 1.5 kJ·mol^−1^) and follows a nucleation-type model (x^1/3^), suggesting that after the initial structural breakdown, decomposition proceeds through localized nucleation and growth [14,15]. These results are consistent with the flexible molecular structure of fenbendazole and the presence of weaker C–S bonds, which facilitate early-stage decomposition [16].

In the case of FLU, the main decomposition process contributes 84.9% of the total probability and displays a higher activation energy (240.0 ± 11.5 kJ·mol^−1^) compared to FEN, indicating enhanced thermal stability. The same decelerating model (1−x)^1/10^ suggests a similar overall reaction mechanism but occurring at higher energy levels. The second process (λ = 12.4%) has a comparable activation energy (238.2 ± 3.3 kJ·mol^−1^) and follows a nucleation-controlled model (x^1/3^). The narrow difference between the two activation energies indicates a more uniform decomposition pathway. The increased stability can be attributed to the fluorinated aromatic substituent, which strengthens intermolecular interactions and increases molecular rigidity.

MEB exhibits the highest thermal stability, with the primary decomposition process accounting for λ = 93.6% and an activation energy of 281.5 ± 11.9 kJ·mol^−1^. The reaction model (1−x)^1/2^ indicates a strongly decelerating mechanism, consistent with a rigid molecular framework and strong intermolecular interactions such as hydrogen bonding. The secondary process, although contributing only with λ = 4.3%, shows a significantly lower activation energy (239.4 ± 1.1 kJ·mol^−1^) and follows a complex reaction model (x·(1−x)^1/2^), suggesting simultaneous nucleation and growth mechanisms. The very high pre-exponential factors (up to 10^30^ min^−1^) further reflect the high energetic demand required to initiate decomposition.

The average activation energies derived from the NPK method are in good agreement with those obtained using the FWO and FR methods, confirming the reliability of the kinetic evaluation. Slightly lower FR values, particularly for FEN and MEB, can be attributed to the sensitivity of differential methods to experimental noise at high conversion degrees.

The comparative analysis of the thermoanalytical data highlights the influence of chemical substitution on thermal stability and provides relevant information for assessing the behavior of these compounds in pharmaceutical and technological applications.

The NPK average *E_a_* (200.7 kJ mol^−1^) closely aligns with the weighted contributions of the two processes, confirming that FEN undergoes two overlapping kinetic regimes with moderate complexity. The relatively small difference between process 1 and process 2 indicates a smooth mechanistic transition, consistent with the monotonic *E_a_* (α) behaviour observed in the NPK curves.

The α-dependent activation energies obtained for all three materials confirm that their thermal degradation follows complex, multi-step mechanisms rather than a single global reaction pathway. FEN exhibits the mildest variations in *E_a_*, suggesting a comparatively simple decomposition process dominated by progressive bond cleavage and the formation of increasingly reactive fragments.

In contrast, FLU displays a non-monotonic *E_a_* profile, which is particularly informative. The initial rise in *E_a_* at low *α* suggests the involvement of an induction or structural rearrangement stage, followed by a main decomposition regime represented by the mid-α plateau. The subsequent decrease at higher conversions implies the formation of low-energy intermediates or diffusion-assisted degradation processes. Such behaviour is typical for polymers or organic solids with overlapping consecutive reactions.

MEB demonstrates the highest degree of kinetic complexity. Its very high initial activation energies highlight a structurally robust network, requiring significant energy input for the primary decomposition steps. The continuous decrease in *E_a_* indicates that once these initial barriers are overcome, the material transitions to increasingly reactive intermediates. The strong divergence between FWO and FR at high α further supports the involvement of autocatalytic phenomena, secondary char formation, or phase changes.

Overall, the comparison between materials indicates that FEN undergoes the simplest degradation pathway, FLU follows a two- or three-stage mechanism, and MEB displays the most energy-intensive and multi-phasic decomposition process.

## 3. Materials and Methods

### 3.1. Materials

FEN (ID: F0040000), FLU (ID: Y0000138), and MEB (ID: M0215000) were obtained from Sigma-Aldrich (St. Louis, MO, USA), with purities conforming to the European Pharmacopoeia (EP) Reference Standards. The compounds were stored in tightly sealed vials following the supplier’s instructions and were used directly in the experiments, with no additional preparation.

### 3.2. Thermoanalytical Investigations

Thermoanalytical studies were performed using a Perkin-Elmer DIAMOND instrument (Perkin-Elmer Applied Biosystems, Foster City, CA, USA) to simultaneously record the TG, DTG and HF curves under a dynamic air atmosphere (100 mL·min^−1^) in open aluminium crucibles (85 μL). The mass of the samples used in the TG/DTG experiments ranged between 1.7 and 3.1 mg, ensuring appropriate thermal response and accurate measurement of mass changes during the analysis. The composition of the synthetic air was 20% oxygen (O_2_) and 80% nitrogen (N_2_). Temperature calibration was carried out using certified reference materials with well-defined melting points (e.g., indium and zinc). The heat flow calibration was performed based on the known enthalpy of fusion of indium. Measurements were conducted under non-isothermal conditions at four heating rates (β = 7, 12, 15 and 20 °C·min^−1^) from room temperature up to 500 °C. Differential thermal analysis (DTA) signals (µV) were converted to heat flow (HF, mW).

Differential scanning calorimetry (DSC) analysis was performed using a NETZSCH DSC 204 F1 Phoenix instrument (NETZSCH, Selb, Germany). Temperature calibration was performed using high-purity reference standards (indium, tin, and zinc) with certified melting temperatures. Enthalpy calibration was achieved using the known heat of fusion of indium. Approximately 5.0 mg of sample was placed in a pierced aluminium crucible (40 μL) and heated from 25 to 300 °C at a heating rate of 10 °C·min^−1^ under a nitrogen atmosphere, using an empty aluminium pan as reference. Each measurement was performed in triplicate to ensure reproducibility.

### 3.3. Kinetic Study

Kinetic data analysis was performed for the first decomposition stage of FEN, FLU, and MEB using the ASTM E698 [17], Friedman [18], and Flynn–Wall–Ozawa [19,20] approaches. The calculations were carried out with AKTS—Thermokinetics Software, version 4.46 (AKTS AG TechnoArk, Siders, Switzerland).

Non-Parametric Kinetics (NPK) is a model-free kinetic analysis method originally proposed by Nomen, Serra, and Sempere [21,22] for the study of complex solid-state reactions, particularly when the reaction mechanism is unknown [23]. Unlike conventional parametric approaches, NPK does not assume a predefined functional form for the dependence of the reaction rate on temperature or conversion. The method is based on Singular Value Decomposition (SVD) [24], which enables the mathematical separation of temperature and conversion effects on the reaction rate. This allows the evaluation of kinetic separability and the validity of the single-step kinetics approximation without prior mechanistic assumptions. Despite its high theoretical accuracy, the application of NPK is limited by its mathematical and computational complexity [25].

The kinetic parameters were expressed using the notation commonly adopted by the ICTAC committee [26,27], where α represents the conversion degree, t—the time, β—the linear heating rate (°C·min^−1^), A—the Arrhenius pre-exponential factor (min^−1^), f(α)—the differential conversion function, g(α)—the integral conversion function, *E_a_*—the activation energy (kJ·mol^−1^), R—the universal gas constant (J·mol^−1^·K^−1^), T—the absolute temperature (K), m_0_—the initial sample mass, m_f_—the final mass after decomposition, and m_T_—the sample mass at temperature T.

## 4. Conclusions

The thermoanalytical data indicate that all three benzimidazole derivatives undergo multi-step thermal degradation, with fenbendazole showing the highest overall mass loss and the most intense high-temperature decomposition, flubendazole presenting intermediate stability, and mebendazole displaying the most moderate and gradual degradation profile. These differences reflect variations in their structural substituents and highlight mebendazole as the thermally most stable compound within the series.

The kinetic evaluation using the FWO and FR isoconversional methods demonstrates clear mechanistic differences among the three API. FEN exhibits a moderately decreasing activation energy, indicative of a relatively straightforward degradation sequence. FLU presents a characteristic non-monotonic *E_a_* profile, suggesting overlapping kinetic regimes and structural transformations during decomposition. MEB shows the highest initial values and the steepest overall decline, reflecting its superior thermal stability but also the presence of multiple, energetically distinct degradation steps. These findings underline the importance of isoconversional analysis for accurately assessing the thermal behaviour and stability of complex materials. The results also offer valuable insight for predicting processing behaviour, selecting optimal temperature windows, and designing materials with tailored thermal performance. The differences observed in the kinetic parameters can be rationalized by considering the structural features of the three benzimidazole derivatives. Although fenbendazole (FEN), flubendazole (FLU), and mebendazole (MEB) share the same benzimidazole core, their substituents significantly influence their thermal behaviour. Fenbendazole contains a thioether bridge (–S–), introducing a highly polarizable atom and relatively weaker C–S bonds, which facilitates bond cleavage and contributes to a smoother degradation pathway reflected by the moderately decreasing activation energy. In the case of flubendazole, the presence of a fluorine substituent on the aromatic ring modifies the electronic distribution and increases the complexity of intermolecular interactions, which may explain the non-monotonic *E_a_* profile and the overlapping kinetic regimes observed during decomposition. Mebendazole, lacking highly polarizable atoms and possessing a more compact molecular structure, exhibits higher initial activation energies and a more gradual degradation behaviour, indicating greater intrinsic thermal stability. These observations demonstrate that the nature of the substituents and the resulting electronic and structural effects play a key role in determining the thermal decomposition pathways and kinetic behaviour of these benzimidazole-based APIs.

The NPK analysis revealed that the thermal decomposition of all three APIs proceeds through at least two kinetically distinct processes, characterized by different contributions, kinetic parameters, and reaction models. The apparent activation energies obtained by the NPK method are in good agreement with those calculated using the isoconversional FWO and FR approaches, confirming the consistency and reliability of the model-free NPK analysis. Overall, the obtained kinetic parameters may provide a useful basis for the potential prediction of processing behaviour and thermal stability of the investigated compounds. However, additional calculations would be required to enable quantitative technological stability assessments.

## Figures and Tables

**Table 1:** Results of the thermal investigations for the three active pharmaceutical ingredients.

Sample	TG		Δ*m*/%	DTG	HF
	T_onset_/°C	T_offset_/°C		T_peak_/°C	T_peak_/°C
**FEN**	197	276	13.58	209, 222	211, 286, 322, 404
	276	365	12.20	328	
	365	500	41.83	404	
**FLU**	205	248	11.64	238	237, 282, 342
	248	290	3.56	272	
	290	500	15.25	343	
**MEB**	185	218	8.41	210	210, 234, 249, 334
	218	280	8.63	236	
	280	500	21.42	335	

**Table 2:** Temperature range for the selected processes as a function of the heating rate used in the kinetic analysis.

β/ °C min^−1^	The Temperature Range for the Selected Process/°C		
	FLU	FEN	MEB
**7**	203–249	192–216	187–219
**12**	217–260	193–224	192–222
**15**	217–260	193–225	194–224
**20**	217–261	194–227	197–231

**Table 3:** *E_a_* values vs. conversion degree α obtained by the two isoconversional methods and the mean value for the analysed process.

*E_a_* (kJ∙mol^−1^) vs. α for						
	FLU		FEN		ME	
α	FWO	FR	FWO	FR	FWO	FR
**0.05**	226.7 ± 4.8	278.5 ± 0.9	227.9 ± 5.4	204.6 ± 7.9	295.6 ± 8.6	359.1 ± 11.3
**0.10**	237.7 ± 3.4	259.5 ± 1.5	224.1 ± 3.0	200.0 ± 2.5	307.3 ± 10.4	349.5 ± 13.9
**0.15**	240.8 ± 3.2	252.7 ± 2.8	221.3 ± 2.4	200.1 ± 2.2	311.0 ± 10.0	321.2 ± 6.6
**0.20**	242.0 ± 3.0	251.4 ± 2.6	218.9 ± 2.1	201.3 ± 1.9	310.6 ± 8.7	301.4 ± 5.0
**0.25**	242.9 ± 2.8	250.7 ± 1.9	217.5 ± 1.9	198.2 ± 1.5	308.6 ± 7.6	284.9 ± 4.1
**0.30**	243.5 ± 2.6	250.6 ± 1.4	216.3 ± 1.8	205.2 ± 1.0	305.3 ± 6.5	271.9 ± 3.9
**0.35**	244.3 ± 2.3	253.0 ± 0.9	215.2 ± 1.7	196.8 ± 0.5	300.9 ± 5.6	255.5 ± 4.1
**0.40**	244.9 ± 2.1	253.3 ± 1.1	214.0 ± 1.6	196.2 ± 0.5	295.9 ± 4.8	247.4 ± 3.8
**0.45**	245.2 ± 1.9	247.3 ± 1.1	213.0 ± 1.5	195.5 ± 0.9	290.5 ± 4.1	229.6 ± 4.0
**0.50**	245.1 ± 1.8	244.5 ± 1.4	211.8 ± 1.4	185.0 ± 2.2	284.6 ± 3.5	219.9 ± 4.4
**0.55**	244.6 ± 1.6	241.5 ± 1.7	210.3 ± 1.3	182.0 ± 3.0	278.3 ± 3.0	207.0 ± 5.0
**0.60**	243.9 ± 1.6	235.0 ± 1.9	208.7 ± 1.3	174.7 ± 4.6	271.7 ± 2.5	199.1 ± 5.6
**0.65**	242.6 ± 1.5	227.9 ± 2.1	206.7 ± 1.3	167.2 ± 6.8	264.8 ± 2.2	190.7 ± 6.3
**0.70**	240.7 ± 1.4	218.6 ± 2.2	204.3 ± 1.3	161.0 ± 9.1	257.8 ± 2.0	186.4 ± 7.1
**0.75**	237.9 ± 1.4	205.5 ± 2.0	201.6 ± 1.4	152.5 ± 11.3	250.5 ± 1.9	179.7 ± 8.0
**0.80**	234.3 ± 1.4	193.9 ± 2.1	198.4 ± 1.5	150.8 ± 11.6	243.1 ± 1.9	176.2 ± 7.9
**0.85**	229.2 ± 1.3	177.3 ± 1.9	194.6 ± 1.7	141.0 ± 13.7	235.1 ± 1.9	170.6 ± 9.8
**0.90**	222.5 ± 1.2	164.2 ± 1.6	190.0 ± 2.0	141.3 ± 12.0	226.3 ± 2.1	161.1 ± 10.9
**0.95**	213.1 ± 1.2	153.2 ± 2.2	184.3 ± 2.3	135.0 ± 9.7	215.3 ± 2.5	159.6 ± 10.6
$\bar{E}$ **_a_** **(kJ∙mol^−1^)**	238.0 ± 10.1	229.4 ± 7.9	209.4 ± 9.4	178.3 ± 30.6	276.5 ± 24.2	235.3 ± 33.0

**Table 4:** The NPK analysis for the three APIs decomposition.

Sample	Process	λ/%	E/kJ mol^−1^	A/min^−1^	n	m	R^2^	f(α)	$\bar{E}$_a_ (kJ mol^−1^)		
									NPK	FWO	FR
FEN	1	82.8	212.4 ± 9.2	1.2·10^23^ ± 1.1·10^5^	1/10	0	0.970	(1−x)^1/10^	200.7 ± 7.8	209.4 ± 9.4	178.3 ± 30.6
	2	13.8	179.8 ± 1.5	1.3·10^19^ ± 7.6·10^4^	0	1/3	0.985	x^1/3^			
FLU	1	84.9	240.0 ± 11.5	6.8·10^24^ ± 7.7·10^5^	1/10	0	0.960	(1−x)^1/10^	233.3 ± 10.2	238.0 ± 10.1	229.4 ± 7.9
	2	12.4	238.2 ± 3.3	1.1·10^24^ ± 1.5·10^11^	0	1/3	0.978	x^1/3^			
MEB	1	93.6	281.5 ± 11.9	4.3·10^30^ ± 6.3·10^5^	1/2	0	0.974	(1−x)^1/2^	273.9 ± 11.2	276.5 ± 24.2	235.3 ± 33.0
	2	4.3	239.4 ± 1.1	2.8·10^25^ ± 4.2·10^11^	1/2	1	0.991	x·(1−x)^1/2^			

## Data Availability

Raw data are available upon request from the corresponding author of this work.
